# Understanding Chrononutrition for Managing Shift Workers' Health: A Narrative Review and Future Directions

**DOI:** 10.5694/mja2.70324

**Published:** 2026-10-20

**Authors:** Yan Yin Phoi, Maxine P. Bonham, Jillian Dorrian, Michelle Rogers, Alison M. Coates

**Affiliations:** ^1^ School of Allied Health and Human Performance Adelaide University Adelaide South Australia Australia; ^2^ Department of Nutrition, Dietetics, and Food Monash University Notting Hill Victoria Australia; ^3^ School of Psychology Adelaide University Magill South Australia Australia; ^4^ School of Public Health Adelaide University Adelaide South Australia Australia; ^5^ Graduate Research School Adelaide University Adelaide South Australia Australia

**Keywords:** circadian rhythm, nutrition, obesity, occupational diseases, occupational health

## Abstract

Shift workers have temporal eating patterns characterised by irregular food intake and prolonged periods of eating with more energy intake occurring in night‐time hours during night shifts. These patterns of eating against a backdrop of shift work exacerbate circadian misalignment, which impairs cardiometabolic profiles and increases the risk of developing overweight and obesity, diabetes and cardiovascular diseases. Limiting food intake at night and being mindful about food choices is beneficial on night shifts, but further research is required to determine appropriate timing and composition of food intake. Some food intake at night may be necessary in supporting individual alertness and performance at work and should be considered when recommending that shift workers minimise food intake at night. At the individual level, shift workers may benefit from minimising night‐time food intake during night shifts. At the organisational level, healthy food consumption habits should be encouraged by improving access to healthy food at night, implementing scheduled meal breaks and providing information about healthy eating guidelines to employees. Medical professionals and Accredited Practising Dietitians (APD) should be aware of the impact of circadian misalignment on metabolic health and consider this when educating patients on adhering to healthy eating guidelines.

## 1 Introduction

Shift workers work outside the conventional hours of 08:00 h and 18:00 h [1], which may include permanent shifts or combinations of rotating evening, night, split, on‐call or 24‐h shifts [2]. In 2023, 14.5% of employees in Australia usually performed shift work [3], with the main industries including mining, healthcare and accommodation and food services [4]. Compared with day‐working counterparts, shift workers have a greater risk of diabetes (14% higher risk) [5], cardiovascular diseases (17% higher risk) and coronary heart disease morbidity (26% higher risk) [6], with night shift work being classified as ‘probably carcinogenic to humans’ by a working group at the International Agency for Research on Cancer [7]. Although the health risks for shift workers may be attributed to lifestyle factors such as smoking or social and domestic stress [8], there are pathways to disease development that stem solely from being awake at night, a time typically associated with rest and recovery.

This is problematic because for shift workers, work hours occur when the body should be at rest, as dictated by our internal circadian rhythm, which is regulated by a central clock in our suprachiasmatic nucleus. This clock oscillates in approximate 24‐h cycles, kept in synchrony with the outside world through the light/dark information transmitted to the clock through our eyes [9]. The central clock coordinates with peripheral clocks located in organs throughout the body through hormonal and neuronal signals to determine our sleep/wake cycle, feed/fast cycle and regulation of physiological processes [9]. For example, metabolic processes (such as insulin secretion, glucose metabolism, diet‐induced thermogenesis and lipid metabolism) peak in the day and decline at night, with late night food consumption resulting in perturbations to metabolic profiles [10, 11, 12, 13, 14, 15, 16]. Additionally, night shifts cause the sleep/wake cycle and feed/fast cycle (internal biological clock) to desynchronise with the 24‐h light/dark cycle, resulting in circadian misalignment [17, 18]. Circadian misalignment leads to impaired glucose tolerance and raised levels of inflammatory markers and blood pressure in both healthy adults [19, 20, 21] and long‐term shift workers (who performed ≥ 12 months of shift work) [22, 23]; with chronic circadian misalignment linked with an increased risk of diabetes, overweight and obesity and metabolic syndrome [24, 25, 26, 27].

There is a growing interest in the cardiometabolic health effects of the temporal pattern of food intake over the 24‐h day/night cycle, known as chrononutrition [28], which holds relevance for night shift workers [28]. Temporal aspects of food intake that impact cardiometabolic health outcomes include duration of eating window (DEW), timing, frequency and regularity of food intake (Figure 1) [28; 29]. Previous reviews before 2020 of shift workers have described their eating patterns and associated health risks [30, 31, 32], reasons behind their dietary choices [32, 33] and dietary interventions [34]. However, the reviews do not consider advantages from, or barriers to, changing food timing, nor include performance outcomes, which may be affected by changing food intake across a shift. This information is critical, not only for shift workers, but also for organisations and healthcare professionals to optimise workplace health promotion and medical chrononutrition therapy and to highlight research gaps to advance the field of chrononutrition.

**FIGURE 1:**
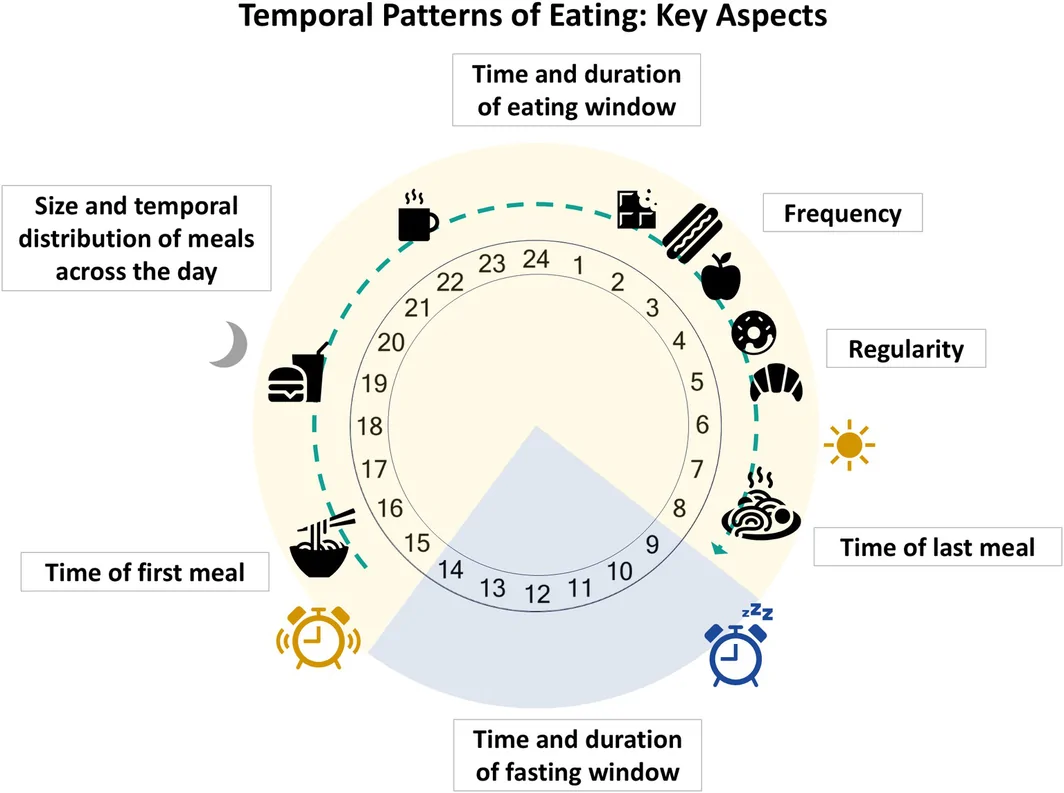
Key temporal aspects of food intake known to impact cardiometabolic health outcomes.

This narrative review aims to provide an update of recent evidence since 2020 and (i) characterise the temporal eating patterns of shift workers across different shift types to highlight their eating patterns; (ii) discuss and evaluate relationships between timing of food intake, and the health and performance outcomes in observational and intervention studies on shift workers or in simulated shift work; and (iii) summarise translational potential for clinical practice and future directions in the chrononutrition management of shift worker health.

## 2 Search Strategy

A search was conducted on MEDLINE, Embase and APA PsycInfo using keywords related to “shift work”, “shift schedule”, “chrononutrition”, “temporal patterns of food intake” (such as timing, frequency and regularity). The search was limited to articles published from 2020 to 2025, written in English, including adult human participants (aged 18 years and older) and excluded animal studies.

## 3 Temporal Eating Patterns of Night Shift Workers

A series of chrononutrition terms were identified in a previous Delphi study as important concepts for examination among shift workers, including DEW, distribution of food intake across different shift types and days off and frequency of food intake [29].

### 3.1 Duration of Eating Window

The DEW is the time between first and last instance of energy consumption, within an individual's wake window [29]. Due to extended wake times, the DEW may differ between night shift workers and those in non‐shift work roles and may further be influenced by shift schedule (although data are limited). For example, a longer DEW was reported in Brazilian police undertaking night shift work compared with day‐working counterparts because of the later timing of the last meal [35]. Whereas in a group of rotating‐shift military police, it was the morning shift, rather than the night shift or day off, that was associated with the longest DEW due to an earlier timing of the first meal [36]. However, these results may not reflect the true eating window around a night shift, as food intake was only captured over a 24‐h period and may have missed any eating occasions before or after the first night shift, which tends to occur within an extended wake window outside the 24 h data capture period as a consequence of missing a night's sleep [37]. Other studies, however, have been more mindful and have measured food consumed within a full wake window (from when participants wake from their main sleep to when they fall asleep for their next main sleep) [37, 38]. When the DEW was examined within a wake window (instead of only 24‐h), two small studies of male, mining, rotating shift workers [38] and night shift workers from mixed industries in Australia [37] reported that the DEW was the longest on the first night shift (day leading into the first night shift) and shortest on the day after the last night shift compared with other shifts/off days. However, on subsequent night shifts (the second night shift onwards in a series of night shifts), the DEW was no different compared with morning shifts [37]. Not surprisingly, the longer DEW on night shifts translated to significantly shorter fasting periods when compared with other shift types among miners in Brazil (intra‐individual comparisons) [39] and with nurses in America [40] and military police officers in Brazil [35] working day shifts (inter‐individual comparisons).

### 3.2 Energy Distribution Over 24 h

Night shift work is also characterised by an altered distribution of energy intake over the 24‐h period. Police officers in Brazil had the greatest energy consumption between 00:00 h and 03:59 h [35] and police officers in Canada had the latest average timing of energy consumption at 23:08 h on night shifts compared with other shifts [41]. A small study in nurses and midwives reported two peaks in energy intake: one between 23:00 h and midnight, and again between 03:00 h and 05:00 h [42]. Shift workers from mixed industries in Australia had later times of first, last and largest eating occasions on night shifts compared to morning shifts [37]. When all eating occasions across 24‐h periods have been considered, studies in Australia and other countries have found a greater dispersion of food intake around the 24‐h clock on night shifts, consistent with longer DEW on those shifts, which is a distinct characteristic compared with other shift types and days off, where there is a clear break from food consumption between around midnight to 06:00 h [37, 39, 41].

### 3.3 Eating Frequency

Meal frequency (number of meals a day) was not found to be significantly different between rotating shift workers and permanent day workers in the Netherlands [43], nor between morning shifts, night shifts and days off within rotating shift workers in Brazil and Poland [36, 44]. In Taiwan, hospital nurses who reported fewer meals on evening/night shifts than morning shifts compensated with increased snacking behaviour [45]. Two studies additionally distinguished between meal frequency on first night shifts from subsequent night shifts [37, 39]. In both studies, one in mixed industries in Australia [37] and another in mining in Brazil [39], meal frequency was greater on the first night shift, linked with a longer DEW, compared with days off but no difference when compared with morning shifts. However, the Australian study noted a lower frequency on subsequent night shifts than first night shifts once the night shift cycle was established [37].

In summary, across different countries and industries, night shift workers re‐distribute eating occasions throughout the night and early morning hours. Although the DEW and eating frequency is not greater on night shifts compared with morning shifts, some studies have identified a prolonged eating window in the day leading into the first night shift. The frequent fluctuations in timing of food intake between shift types may result in irregularity in timing of food consumption that is asynchronous with the circadian rhythm.

## 4 Consequences of Food Consumption at Night and Circadian Misalignment

Higher risks of poorer metabolic health, including higher cardiovascular disease risk, are observed in night shift working populations who eat later at night [46, 47] or have a longer DEW [47] compared with individuals who did not, after adjusting for confounders. The mechanisms that contribute to these associations include disturbances in lipid, insulin and glucose profiles, resulting from a combination of circadian misalignment from shift work and mistimed food intake.

A controlled laboratory study demonstrated the nadir in the circadian rhythm of triglyceride metabolism occurred at night and the resultant elevation in triglyceride levels from night‐time food consumption [48]. Similarly, glucose tolerance was poorer when young healthy participants consumed food at night in a simulated shift work study [49] and among permanent night/rotating shift workers in a randomised controlled crossover study comparing high sugar meals consumed at night during a night shift to in the day during a day shift or day off [50]. In contrast, limiting food intake to the daytime during simulated shift work allowed endogenous circadian glucose rhythms to remain unchanged from baseline and synchronised to the central clock rhythm, avoiding glucose intolerance [49]. Additionally, significantly higher overall mean 24‐h insulin levels have been observed for night shift workers than for day shift workers in an observational study, with eating at night associated with more than two times greater insulin levels [51]. No differences in insulin levels between day and night shift workers were observed when eating was primarily in the daytime [51]. This highlights the benefits of eating in the day, in synchrony with the circadian rhythm despite performing shift work.

## 5 Benefits of Limiting Night‐Time Food Intake

To minimise the metabolic consequences of night‐time eating, studies have investigated the potential benefits of avoiding (fasting) or minimising food intake at night on metabolic health outcomes.

The impact of fasting from 22:00 h until 06:30 h during two night shifts, compared with two nights of consuming a meal at 02:00 h, was compared in male police officers. Fasting on night shifts resulted in significantly lower morning insulin levels and insulin resistance, than in the individuals who did not fast [52]. However, the overnight fast resulted in a higher consumption of calories during the subsequent day compared with the individuals who did not fast [52]. Although fasting prevented unwanted increases in glycaemic responses at night, the higher subsequent energy intake could have implications for weight gain if sustained.

Time‐restricted eating (TRE) has become a popular practice for the management of weight and several metabolic conditions [53]. In a 12‐week study (*n* = 137), firefighters who worked 24‐h shifts were randomised to receive either standard of care (SOC; nutritional counselling for the Mediterranean diet) or SOC and 10‐h of TRE (self‐selected eating window) during the day. Although there were no between‐group differences in energy intake or body mass index (BMI), both groups reduced energy intake, and the TRE group had a significant decrease in body weight and BMI. Additionally, post hoc analyses revealed that individuals with higher glycated haemoglobin levels or blood pressure at baseline experienced improvements to those parameters with SOC plus TRE but not SOC alone. The metabolic benefits found in this study were likely due to the rearrangement of energy intake towards daytime hours, and demonstrated that implementing a self‐selected TRE window plus SOC was as effective as using SOC alone and a suitable alternative among shift workers, with suggested benefits accrued by individuals with increased cardiometabolic disease risk [54].

## 6 Considering Practicalities and Addressing Challenges of Fasting at Night

Metabolically, shift workers may benefit from fasting at night, but a qualitative study found that consumption of food during night shifts also serves the purpose of social bonding, improving team moral and maintaining alertness [55]. In a simulated shift work study in Japan, 15 healthy adults were provided a snack at 03:30 h during the night shift and exhibited better cognitive performance than without a snack [56]. Additionally, in shift‐working nurses in the Netherlands, a greater eating frequency was associated with greater alertness, measured by a faster reaction time and a lower number of lapses, although the type or size of meals consumed is not clear [57]. These studies recognise the importance of food intake at night for maintaining work performance, but cardiometabolic health outcomes were not investigated. Maintaining performance is an important factor to consider for shift workers with high workload or where mental acuity is crucial for safety. Hence, some studies have investigated shortening the duration of fasting [58] or introducing the concept of intermittent fasting [59]; while others manipulated the caloric load [60] or frequency of food provided while adjusting its content [61].

Two randomised controlled trials restricted food intake on night shifts to address the challenges of entirely omitting food consumption during night shifts [58, 59]. In a randomised crossover feasibility study in Australia, permanent or rotating night shift workers with abdominal obesity who usually ate between 01:00 h and 06:00 h on night shifts were asked to avoid food consumption during that window for 4 weeks [58]. This resulted in a significant reduction in body weight and BMI, while maintaining similar energy intake between intervention and control periods (habitual food intake) [58].

Although the weight change observed in the previous study was not clinically relevant, it informed the development of a weight loss trial (Shifting Weight using Intermittent Fasting in night shift workers (SWIFt) study) among 250 night shift workers in Australia [59]. The trial examined the effectiveness of modified intermittent fasting (IF) by limiting energy intake to 500 kcal on either day shifts/days off (IF: 2D) or night shifts (IF: 2N) with no restriction on other days, compared with control (continuous energy restriction [CER] of 20% of estimated energy requirements [EER]) [59]. After 24 weeks, weight loss and decreased insulin resistance were observed in all groups, with no significant differences between either IF or CER groups [59]. All groups also experienced significant improvements from baseline in fasting insulin levels, blood pressure, waist circumference, BMI and fat/fat free mass [59]. This study demonstrated three potential strategies for night shift workers to successfully lose weight, with the IF: 2N group providing a mechanism to minimise overnight eating [59]. A qualitative study of a subset of study participants reported that fasting on only 2 days a week was straightforward to follow and easy to integrate into work schedules [62]. Participants did not find fasting at night to be disruptive as it did not interfere with day‐time social activities; however, those whose work schedule required fasting on back‐to‐back days (common for rotating shift workers), reported side effects including headaches, hunger and nausea, that improved with strategies provided by study dietitians [62].

The impact of snacks compared with meals during simulated or actual night shifts has also been tested to determine if caloric load of each instance of food intake at night matters. In a simulated shift work study in Australia, 55 healthy, non‐obese adults were randomised to either fast at night, snack at night (10% of EER), or have a meal (30% of EER) at night (00: 30 h) [60]. Although impairment in glucose tolerance was less after a snack compared with a meal, glucose tolerance was impaired compared with no energy intake at all, highlighting the significance of a fast for glucose homeostasis [60]. The authors noted that altering the macronutrient content of study food to be higher in protein and lower in carbohydrates may lead to different results, requiring further investigation [60]. Another study in the Netherlands also manipulated the frequency (1 meal vs. 3 meals) and content (high glycaemic index (GI) vs. low GI) of food consumed at night [61]. In comparison with not having meals during night shifts, consuming low GI meals led to less hunger and did not worsen glucose homeostasis, regardless of meal frequency [61, 63]. Congruently, consuming high GI meals, regardless of frequency, led to worse glucose variability metrics. Low GI meals also reduced the number of lapses in a psychomotor vigilance test compared with high GI meals [63]. Incorporating low GI food options is a simple approach that could limit glycaemic impairment while supporting alertness associated with shift work [63].

Data in this review support found that consumption of small amounts of food at night maintained work performance. Previous studies (published before the timeframe of this review) found less sleepiness and improved cognitive performance (reaction time, psychomotor vigilance and running memory) with either consumption of a snack or meal compared with food avoidance [64, 65]. What is less clear is when and what to eat, to minimise the impact on metabolic health without compromising work productivity and safety. Future studies need to fill knowledge gaps by examining the concomitant effects of small amounts of food at night on cardiometabolic health outcomes, cognitive performance measures and physiological comfort. Additionally, organisational support is necessary for timely breaks and the provision of healthy food, which is often cited as a barrier [33, 66]. Altogether, these steps are necessary to ensure dietary recommendations to shift workers are not just effective, but also practical and sustainable in the long term.

## 7 Future Directions to Improve Shift Worker Health Through Chrononutrition

Studies from different industries and countries demonstrate consistently that shift workers' food intake is characterised by irregular timing, including food intake throughout the night while working night shifts and prolonged eating windows on first night shifts. Late night food intake impairs glucose and lipid profiles, with long‐term implications of heightened risks of cardiometabolic diseases. These impacts of shift work are different for each shift worker. The short‐term exposure to shift work by rotating medical interns is likely very different to those exposed to chronic shift work. From a chrononutrition perspective, minimising energy intake at night is beneficial in the limited number of studies available, but its long‐term sustainability and outcomes in shift workers with pre‐existing cardiometabolic diseases has yet to be determined (Figure 2). If food is to be consumed for performance and safety, identifying exactly when and what to eat during the night shift with minimal metabolic consequences needs to be further investigated.

**FIGURE 2:**
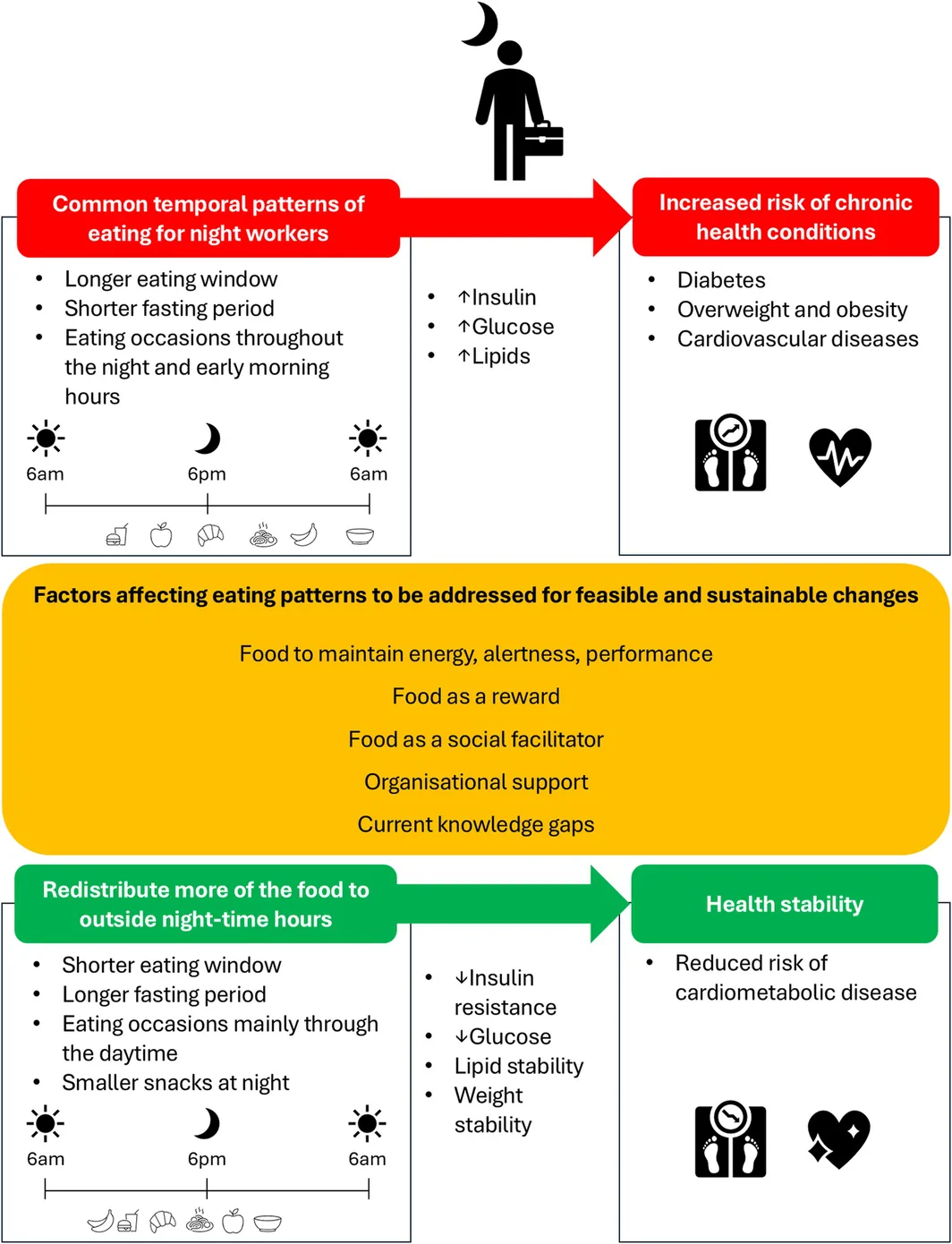
Hypothetical mechanisms describing the link between mistimed food intake among night shift workers and poor health outcomes, with proposed solutions, associated benefits and challenges that should be considered in the future.

Moving forward, improving shift worker health by optimising their diet should be approached from three angles (Table 1). At the individual level, shift workers should be conscious of meal timing and ensure that meal composition is aligned with the Australian Guide to Healthy Eating [67]. Additionally, approaching dietary adjustments collectively with colleagues may make change easier. At the organisational level, employers should support shift workers by removing barriers to healthy eating, consistently highlighted as poor availability of healthy food at night and the lack of time to consume meals or prepare healthy food during shifts [55, 68]. Enablers to eating earlier on night shift have been reported to include scheduled meal breaks and workplace nutritional education programs [66]. A recently developed chrononutrition questionnaire, which captures temporal patterns of eating across different shift types in 10–15 min [29, 69], may be used by organisations in collaboration with Accredited Practising Dietitians familiar with the principles of chrononutrition to design workplace health promotion programs to improve the temporal eating patterns of their unique employee populations. As healthcare professionals, dietitians may additionally provide tailored nutrition education to at‐risk individuals, with referrals to medical professionals or holistic care and follow‐up as necessary [70]. The healthcare system, with the second highest proportion of shift workers of any industry in Australia (32%) and as major providers and advocates of optimal health, should lead shift work industries in supporting its shift‐working employees in consuming timely, healthy food to optimise their health [4].

**TABLE 1:** Strategies to optimise the diet of night shift workers derived from current evidence presented, including elements of the Australian Guide to Healthy Eating, [67] with research gaps that need to be addressed by future studies.

Strategies			Evidence gaps to be addressed by future studies
Individual	When	Limit eating window [54], particularly on first night shift, where eating window tends to be longer [37, 38] Avoid, where possible, or minimise night‐time food consumption [58, 59, 60], considering that prolonged food abstinence at night may cause physiological discomfort [62] and small amounts of food aids alertness and cognitive performance [56, 57]	Differences in temporal eating patterns by shift type and specific dietary improvements required by shift type An appropriate eating window and potentially optimal time(s) of food consumption if small amounts of food need to be consumed during the night shift for cardiometabolic health, cognitive performance and physiological outcomes The impact of limiting food intake at night in individuals with pre‐existing cardiometabolic diseases
	What	Align food choices with the Australian Guide to Healthy Eating [67] Replace high fat takeaway foods with ready to eat balanced frozen meals or hearty soups that are low GI [61, 63] Replace energy dense, low nutrient snacks (e.g., lollies, chips) with low calorie options (e.g., fruits, vegetable sticks) or high protein [60] or high fibre options (e.g., oatmeal, air popped popcorn, trail mix)	The impact of consuming food of different diet compositions (e.g., high fibre, high protein) during the night shift on cardiometabolic health and cognitive performance outcomes
	How	Support colleagues with making healthier choices. Cover colleagues at work and take turns having meal breaks Cook and freeze large batches of healthy food to bring to work Purchase healthy and convenient snacks that are practical to consume at work	
Organisational	Scheduled meal breaks [55, 66, 68] Provide healthy food at work [55, 66, 68] Screening of shift workers' temporal patterns of eating and nutrition education		Cardiometabolic and dietary outcomes in shift workers after chrononutrition intervention by shift work organisations and healthcare professionals.
Healthcare professionals	Understand and implement chrononutrition principles in patient care [28] Screening of shift workers' temporal patterns of eating and nutrition education		

Abbreviation: GI, glycaemic index.

## Author Contributions

Conceptualisation: Yan Yin Phoi, Alison M. Coates; Methodology, Writing – review and editing: Yan Yin Phoi, Maxine P. Bonham, Michelle Rogers, Jillian Dorrian, Alison M. Coates; Data curation: Jillian Dorrian, Yan Yin Phoi; Formal analysis, Investigation, Project administration and Writing – original draft: Yan Yin Phoi; Supervision and Validation: Maxine P. Bonham, Michelle Rogers, Jillian Dorrian, Alison M. Coates; Resources: Alison M. Coates.

## Funding

The authors declare no funding sources.

## Disclosure

Commissioned; externally peer reviewed.

## Conflicts of Interest

The authors declare no conflicts of interest.

## Data Availability

There are no data in this manuscript.
